# Supplementary material for: Hyperactivation of HER2-SHCBP1-PLK1 axis promotes tumor cell mitosis and impairs trastuzumab sensitivity to gastric cancer
Source: Nat Commun. 2021 May 14;12:2812. doi: 10.1038/s41467-021-23053-8 (PMC8121856; doi:10.1038/s41467-021-23053-8)
Supplement: Supplementary file 1 — Supplementary information [file 41467_2021_23053_MOESM1_ESM.docx]

**Supplementary Figures and Tables**

**
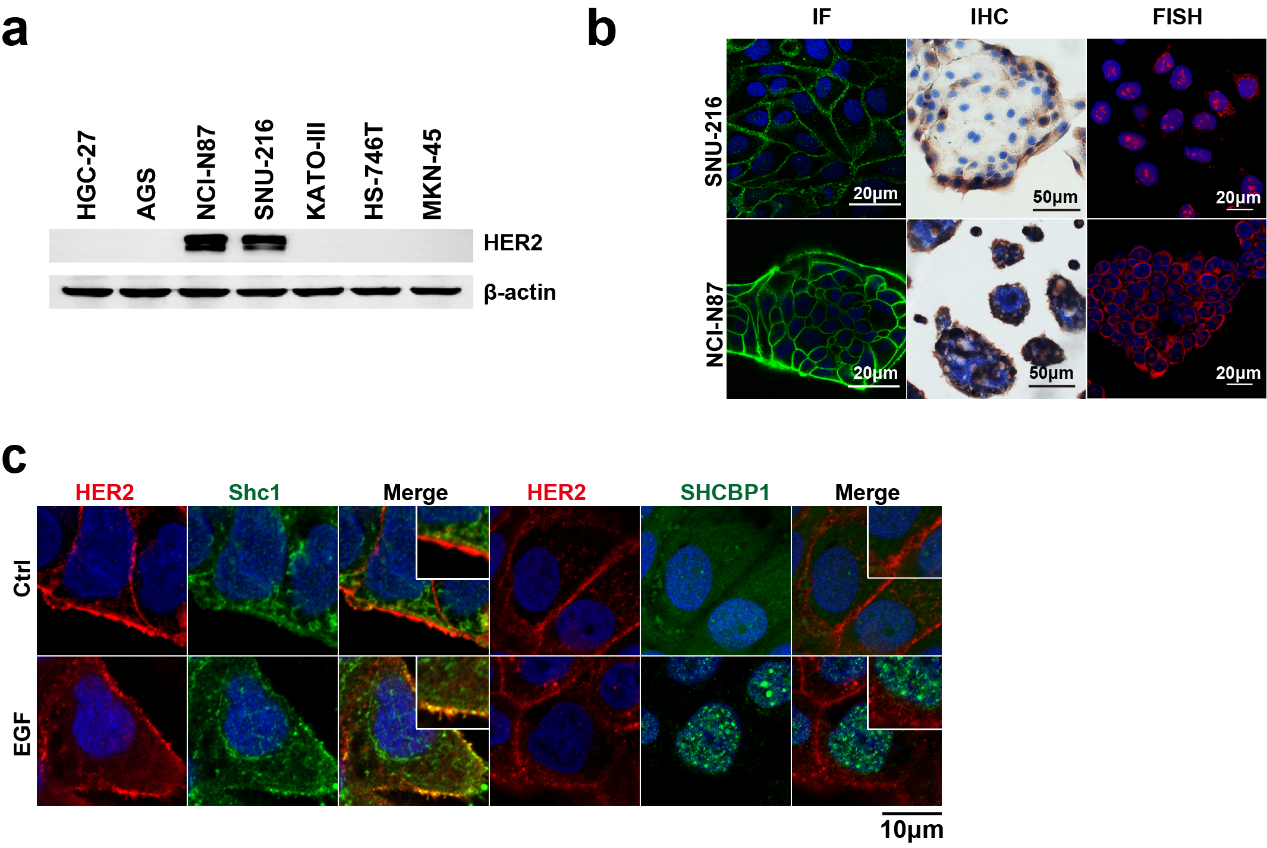
**

**Supplementary Fig. 1 | Identification of HER2 positive gastric cancer cells and HER2 colocalization with Shc1 and SHCBP1. a,** HER2-positive gastric cancer cell lines, NCI-N87 and SNU-216, were identified from 7 gastric cancer cells using immunoblotting. **b,** HER2 expression in NCI-N87 and SNU-216 were confirmed using immunofluorescence (IF), immunohistochemistry (IHC) and fluorescence in situ hybridization (FISH). **c,** Immunofluorescence colocalization of HER2 with Shc1 or SHCBP1 in SNU-216 cells treated with/without EGF. Cells were immunostained with anti-HER2 antibody (red), anti-Shc1 antibody (green), anti-SHCBP1 antibody (green) and DAPI (blue). Data of **a** are representative of two independent experiments. Data of **b** and **c** are representative of three independent experiments.

**
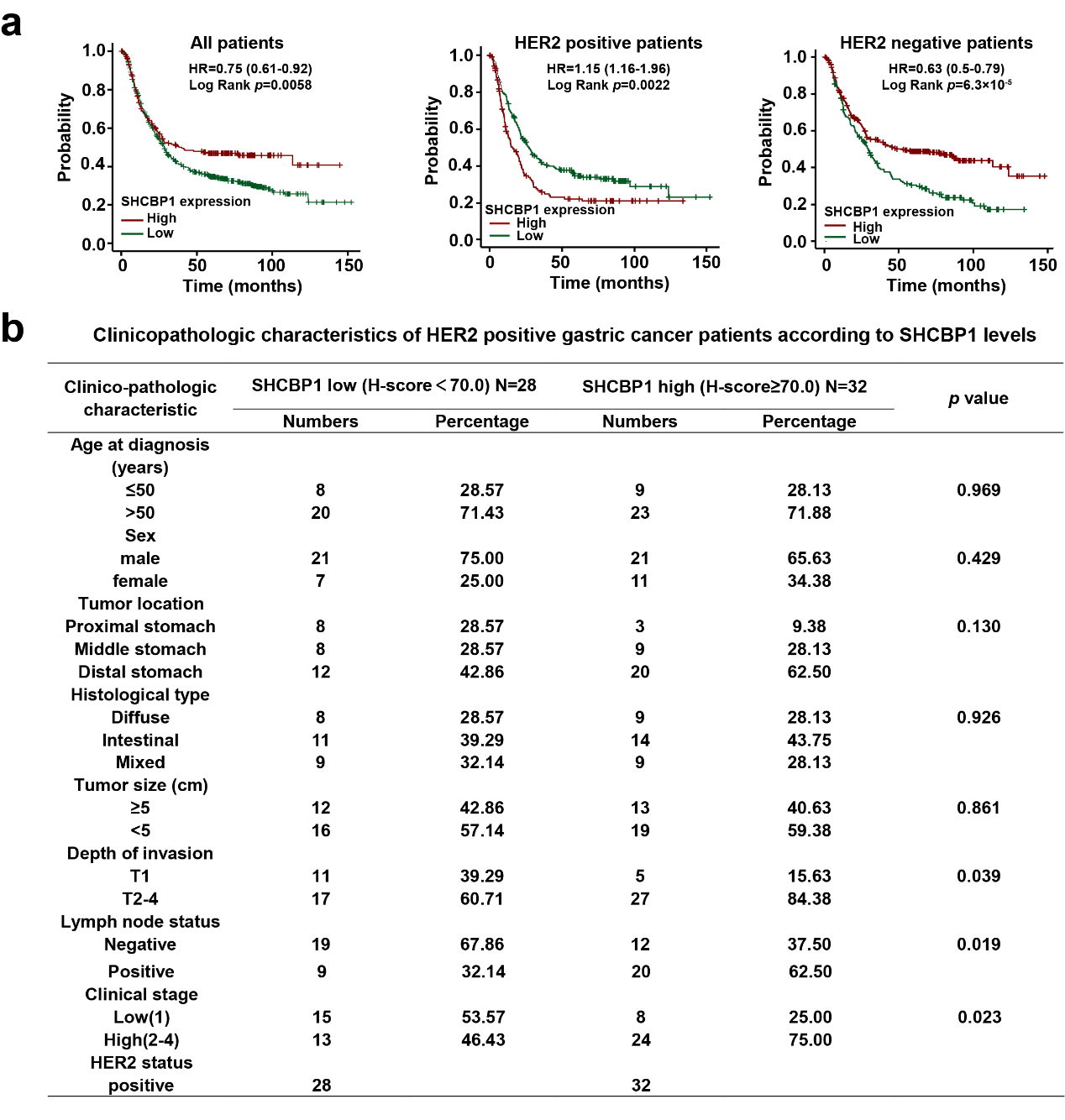
**

**Supplementary Fig. 2 | SHCBP1 correlates with patient overall survival and clinicopathological characteristics.** **a,** Kaplan-Meier plot of the correlation between SHCBP1 expression and patient overall survival for HER2 positive and/or negative patients from GEO database ( <http://kmplot.com/analysis/> ). HR: hazard ratio. **b,** Clinicopathologic characteristics of HER2 positive gastric cancer patients according to SHCBP1 levels from gastric cancer TMAs. H-score: histoscore. The *p* values were determined by two-sided chi-square test (n=60 independent biological samples).


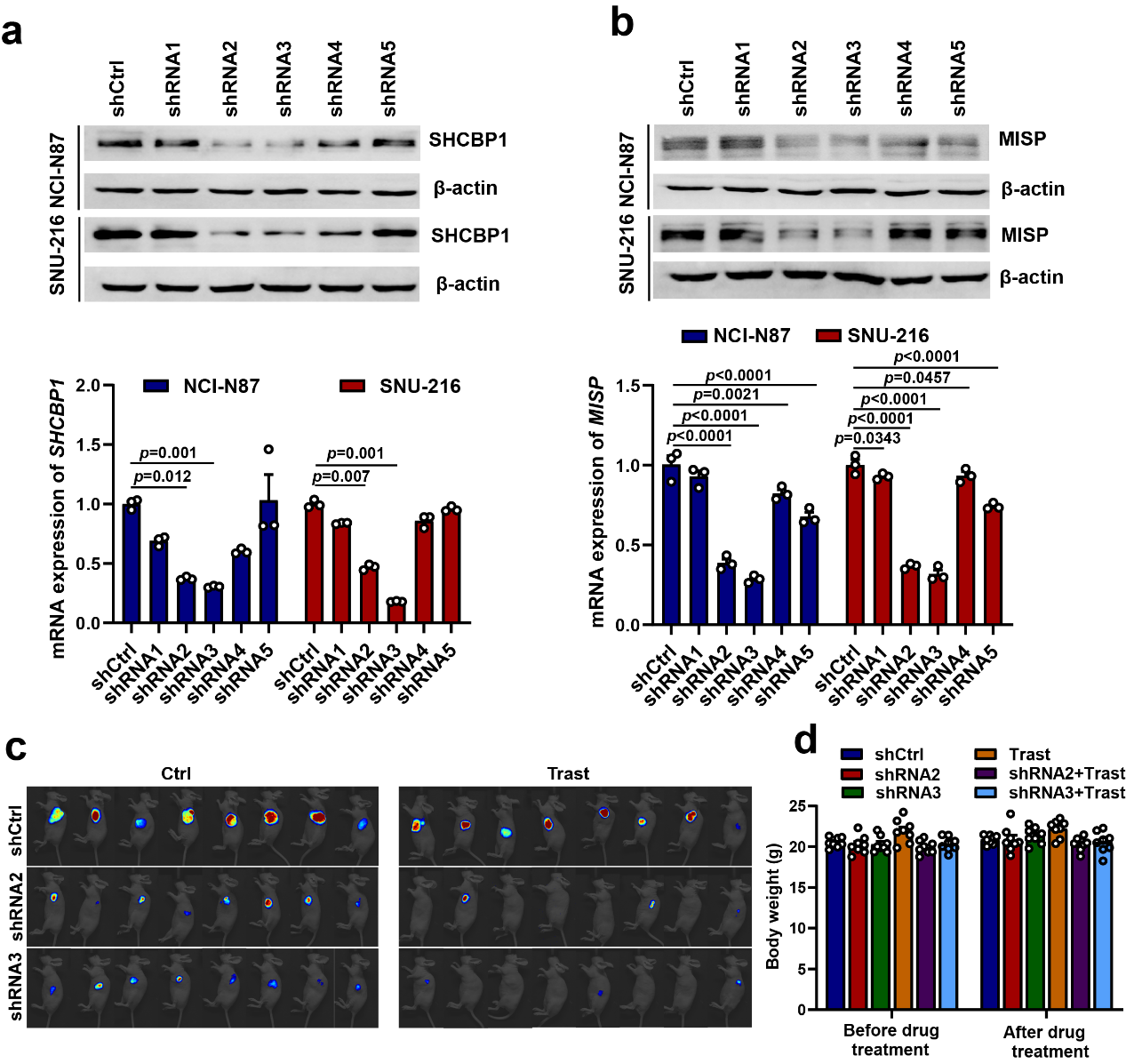


**Supplementary Fig. 3 | Validation of shRNA knockdown efficiency and mice with** **NCI-N87 cell xenografts. a,** Validation of SHCBP1 knockdown in NCI-N87 and SNU-216 cell lines using immunoblotting (top) and real time PCR analysis (bottom). Data are the mean ± s.e.m. The *p* values were determined by two-sided non-parametric test (n=3 independent biological samples). **b,** Validation of MISP knockdown in NCI-N87 and SNU-216 cell lines using immunoblotting (top) and real time PCR analysis (bottom). Data are the mean ± s.e.m. The *p* values were determined by one-way ANOVA (n=3 independent biological samples). **c,** Images of mice carrying shCtrl or SHCBP1 knockdown NCI-N87 cell xenografts after trastuzumab treatment (intraperitoneal; 10 mg/kg; 2 × /wk × 3) for 21 days. **d,** Body weight changes of mice with NCI-N87 tumors before and after trastuzumab treatment. Data are the mean ± s.e.m (n=8 independent mice per group). Western blot of **a** and **b** are representative of two independent experiments.

**
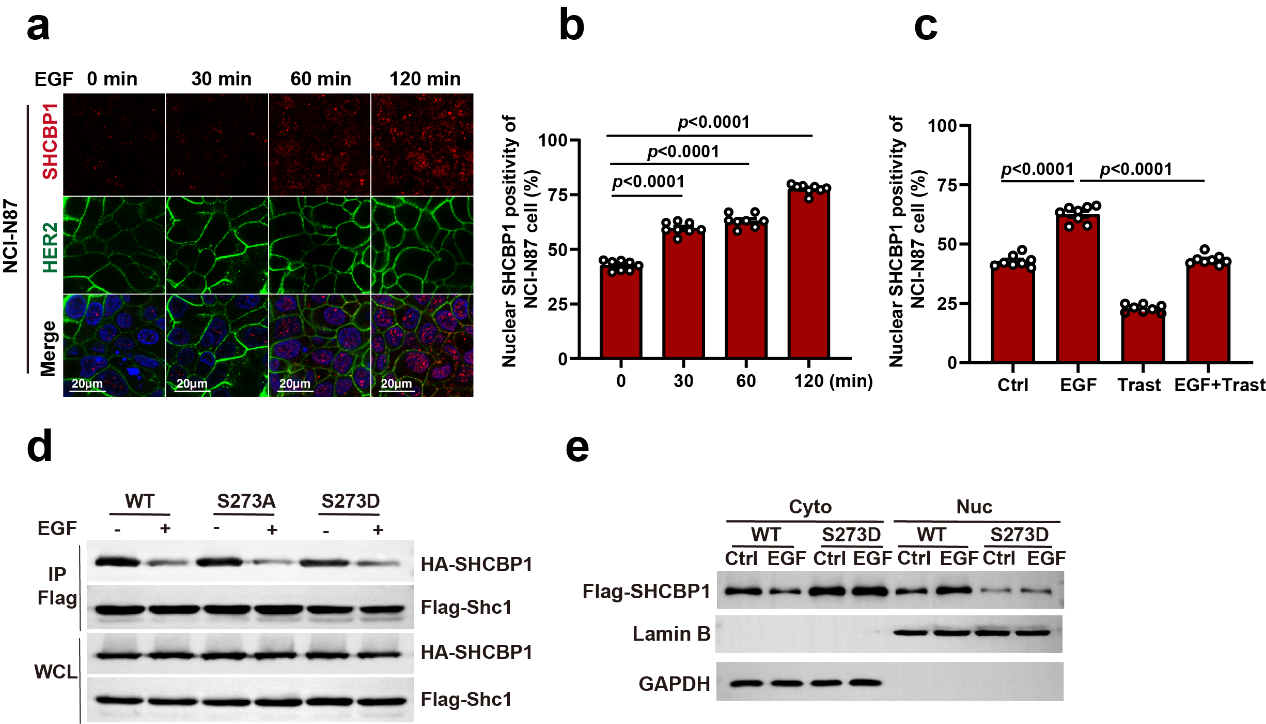
**

**Supplementary Fig. 4 | EGF induces nuclear localization of SHCBP1. a,** Immunofluorescence analysis of SHCBP1 nuclear localization in NCI-N87 cells treated with 100 ng/mL epidermal growth factor (EGF) at the indicated times. Cells were co-stained with anti-SHCBP1 antibody (red), anti-ERBB2 antibody (green), and DAPI (blue). **b,** Nuclear SHCBP1 positivity of NCI-N87 cells following EGF treatment for different times. Eight fields containing at least 50 nuclei were counted in each treatment group. Data are the mean ± s.e.m. The *p* values were determined by one-way ANOVA (n=8 independent biological samples). **c,** Nuclear SHCBP1 positivity of NCI-N87 cells after EGF (100 ng/mL) and/or trastuzumab (Trast, 100 μg/mL) treatment. Six fields containing at least 50 nuclei were counted in each treatment group. Data are the mean ± s.e.m. The *p* values were determined by one-way ANOVA (n=8 independent biological samples). **d,** Co-immunoprecipitation assays of Flag-Shc1 together with HA tagged mutant SHCBP1 (S273A and S273D) in SNU-216 cells treated with 100 ng/mL EGF for the indicated time. WT: wild type; IP: immunoprecipitation; WCL: whole cell lysates. **e,** Nuclear localization detection of Flag-tagged SHCBP1 or mutant SHCBP1 (S273D) using immunoblotting. Data of **a** are representative of at least three independent experiments. Data of **d** and **e** are representative of two independent experiments.

**
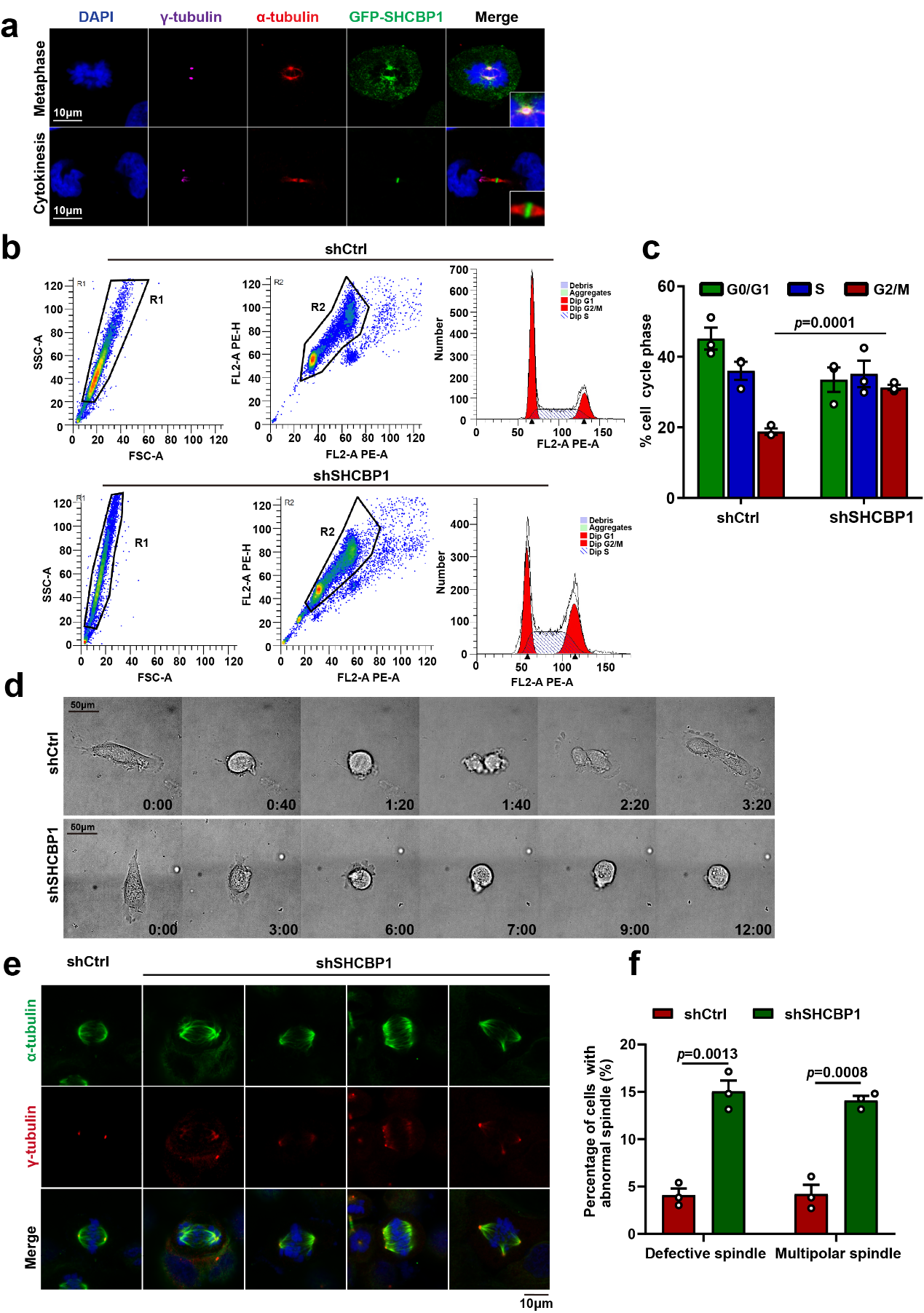
**

**Supplementary Fig. 5 | The effects of SHCBP1 knockdown on cell mitosis of gastric cancer cells. a,** Colocalization of SHCBP1 with spindle and centrosome in metaphase or cytokinesis of mitotic cells. SNU-216 cells stably expressing GFP-tagged SHCBP1 were synchronized using nocodazole blocking, and cells were immunostained with anti-α-tubulin antibody (red) for spindle, anti-γ-tubulin antibody (purple) for centrosome, and DAPI (blue) for DNA. **b and c,** Cell cycle and the statistical results of shCtrl and SHCBP1 knockdown cells detected by flow cytometry. Cells were firstly gated without cell debris (R1) and then gated without adhesion cells (R2). Resulting cells were analyzed for stages G0/G1, S and G2/M. Data are the mean ± s.e.m. The *p* values were determined by two-sided Student's *t*-test (n=3 independent biological samples). **d,** Representative images of time-lapse analysis of SNU-216 shCtrl and SHCBP1 knockdown cells. **e,** Immunofluorescence detection of the mitotic spindle and the centrosome in SNU-216 shCtrl and SHCBP1 knockdown cells. The spindle was stained with anti-α-tubulin (green), centrosome with anti-γ-tubulin (red) and the DNA with DAPI (blue). **f,** The statistical results of the defective spindle and the multipolar spindle in SNU-216 shCtrl and SHCBP1 knockdown cells. Data are the mean ± s.e.m. The *p* values were determined by two-sided Student's *t*-test (n=3 independent biological samples). Data of **a, d** and **e** are representative of three independent experiments.

**
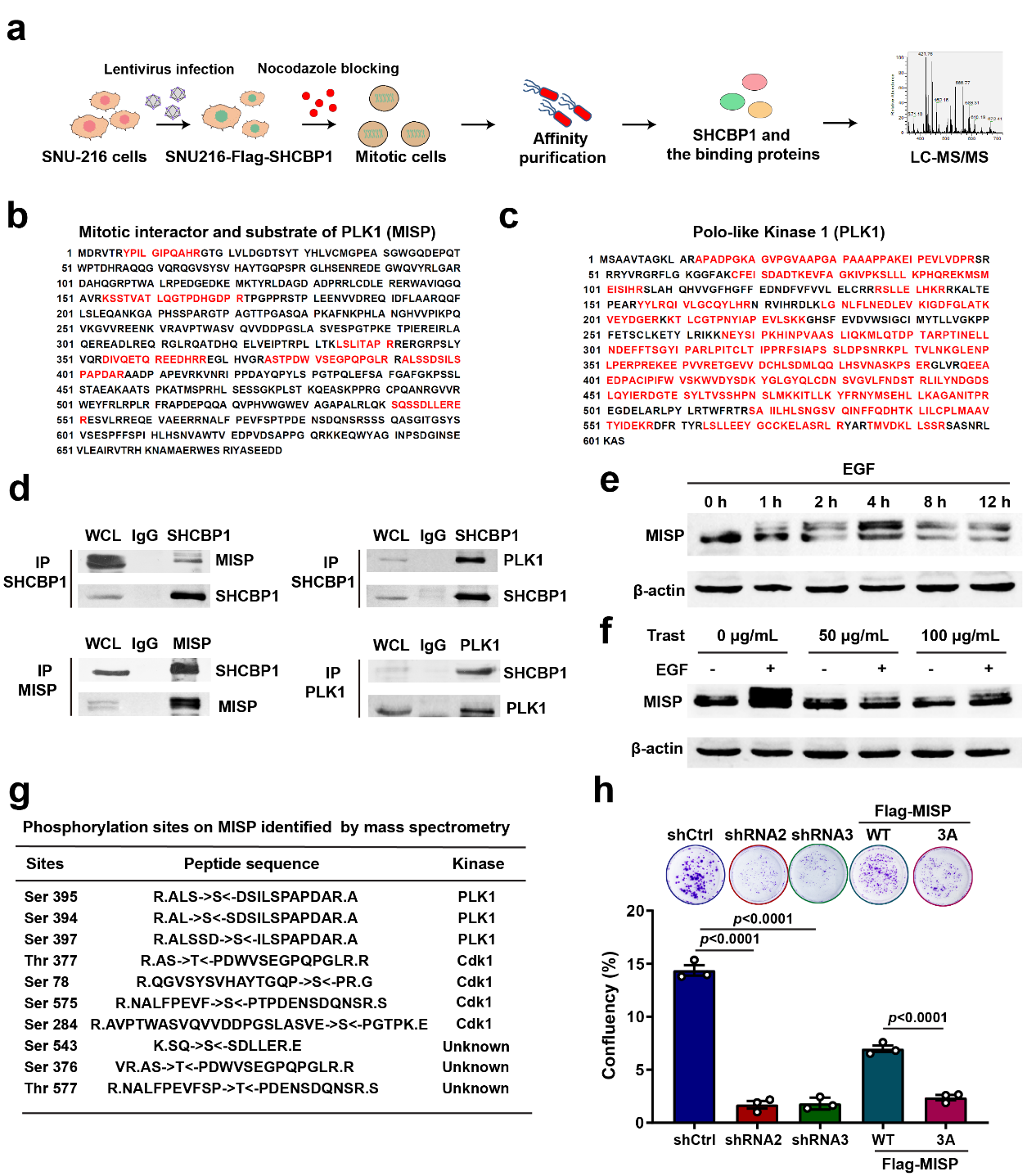
**

**Supplementary Fig. 6 | SHCBP1 binds with PLK1 and MISP for mitotic progression. a,** A schematic representation illustrating the identification of SHCBP1 interactors in the mitotic cells. **b and c,** SHCBP1-interacting proteins MISP and PLK1 in mitotic SNU-216 cells were identified by LC-MS/MS analysis. The identified peptides were indicated in red. **d,** Co-immunoprecipitations of SHCBP1, PLK1 and MISP in nocodazole-arrested SNU-216 cells using rabbit anti-SHCBP1, mouse anti-PLK1, rabbit anti-MISP or IgG antibodies. **e,** Immunoblotting assays of MISP phosphorylation in SNU-216 cells following epidermal growth factor (EGF, 100 ng/mL) treatment at the indicated times. **f,** Immunoblotting assays of MISP phosphorylation in SNU-216 cells following 100 ng/mL EGF and/or trastuzumab (Trast) treatment. **g,** Phospho-peptides of MISP identified by LC-MS/MS analysis in PLK1-overexpressed cells. **h,** Colony formation of SNU-216 cells with two different MISP shRNAs and/or those re-expressing MISP (WT) or mutant MISP (3A); Data are the mean ± s.e.m. The *p* values were determined by one-way ANOVA (n=3 independent biological samples). Data of **d**-**f** are representative of two independent experiments.


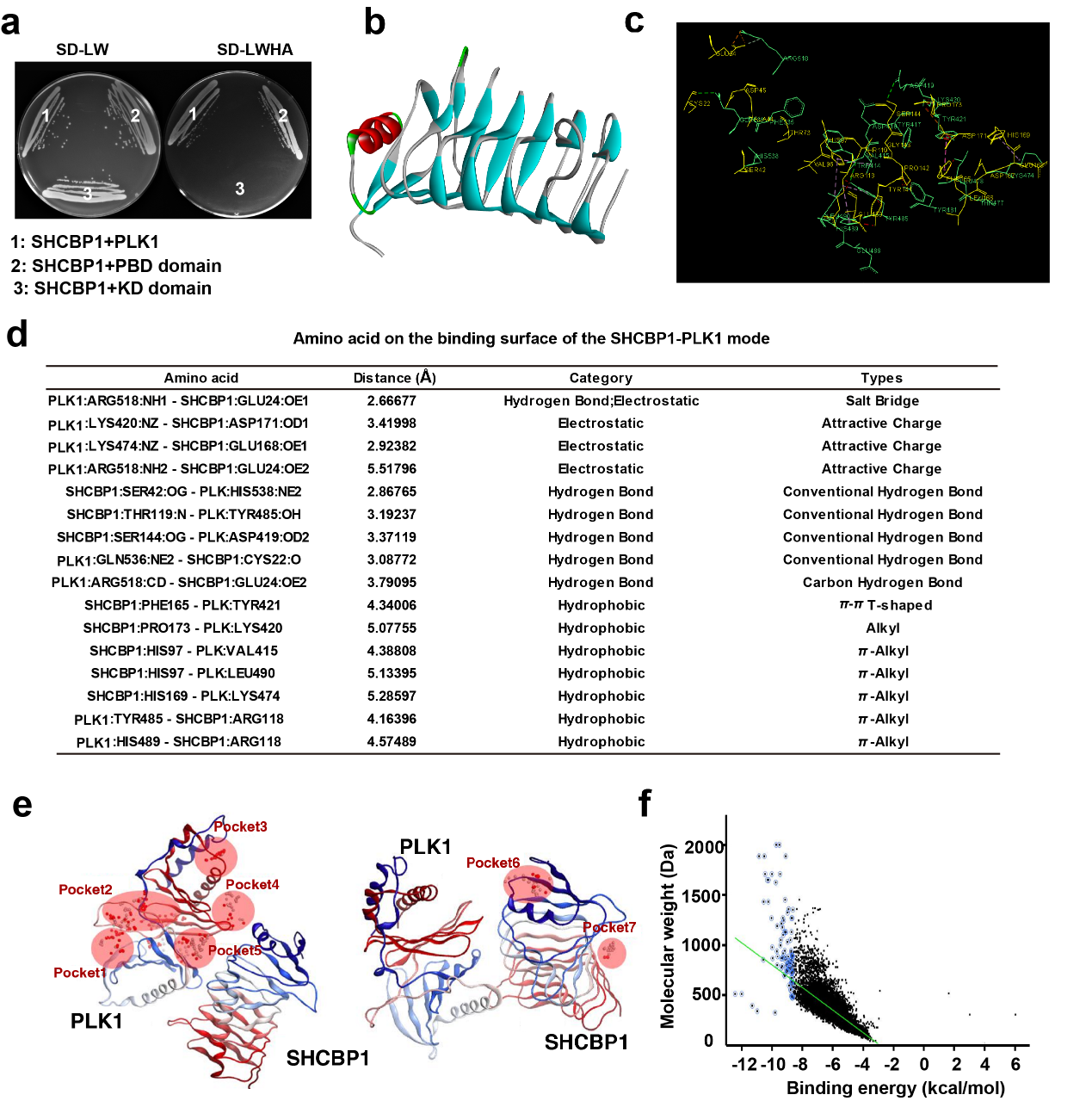


**Supplementary Fig. 7 | The Mode of SHCBP1-PLK1 interaction. a,** Yeast two-hybrid interaction assay of SHCBP1 and kinase domain (KD) or polo-box domain (PBD) of PLK1. SD-LW: Synthetic dropout Leu and Trp; SD-LWHA: Synthetic dropout Leu, Trp, His and adenine. **b,** 3D structure of SHCBP1 355-562 aa domain predicted by homologous modeling with I-TASSER server. **c,** Structural formula and interaction bonds of amino acids on the binding surface of the SHCBP1-PLK1 mode. **d,** A list of amino acids on the binding surface of the SHCBP1-PLK1 mode. **e,** Inhibitor binding pockets on the SHCBP1 355-562 aa domain and PBD domain of SHCBP1-PLK1 mode predicted using MOE-Site Finder plug-ins. **f,** Affinity results of 17,676 small molecules with PLK1 analyzed by Virtual Screening. Selected molecules with binding energy >-8.6 kcal/mol were in blue.


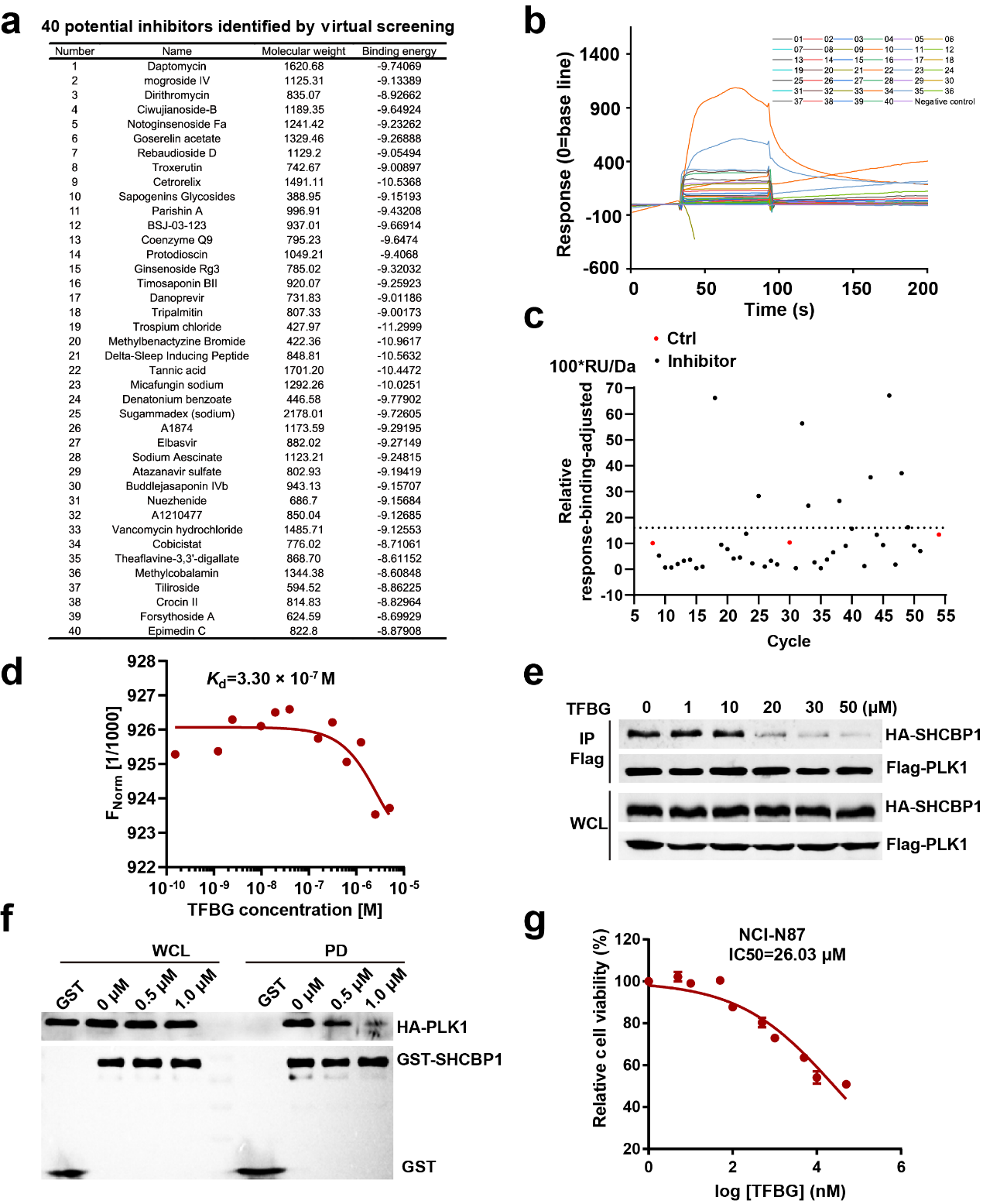


**Supplementary Fig. 8 | Identification of theaflavine-3, 3'-digallate (TFBG) as an Inhibitor of SHCBP1-PLK1 Interaction. a,** 40 potential inhibitors identified by Virtual Screening. **b and c,** Sensorgram and result Plot MW Adjustment of Surface Plasmon Resonance (SPR) screening on the 40 potential inhibitors binding with PLK1. **d,** Kinetic constant (*K*_d_) analyses of theaflavin-3, 3'-digallate (TFBG) interacting with PLK1 using microscale thermophoresis (MST) assays. **e,** Co-immunoprecipitation assays of the PLK1-SHCBP1 interaction in cells treated with TFBG at the indicated concentrations. IP: immunoprecipitation; WCL: whole cell lysates. **f,** Pull-down assays of SHCBP1 and PLK1 interaction following 0.5 μM or 1 μM TFBG treatment. 6His-GST-tagged SHCBP1 and HA-tagged PLK1 expressed in *E. coli* were pulled down and analyzed by immunoblotting. WCL: whole cell lysates; PD: pull-down. **g,** TFBG dose-dependent response in NCI-N87 cells for 4 days. Cell viability was assayed. Data are the mean ± s.e.m (n=6 independent biological samples). Data of **e** and **f** are representative of two independent experiments.

**
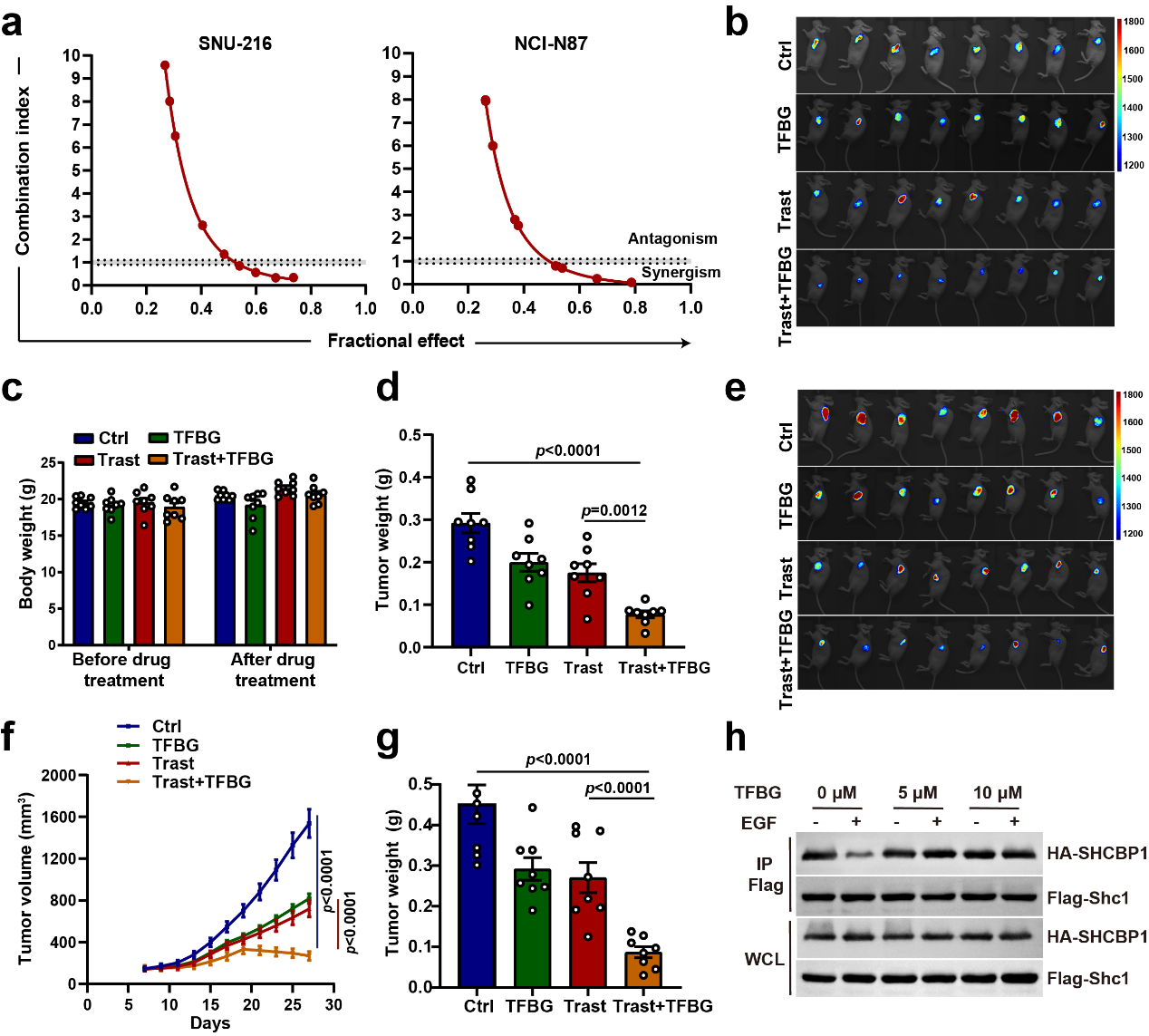
**

**Supplementary Fig. 9 | Combination of TFBG-Trastuzumab efficaciously suppresses the growth of HER2-positive gastric cancer. a,** Analysis of the combined drug effects of TFBG and trastuzumab using the Chou and Talalay method. Synergistic effects are indicated as the combination index (CI) is significantly less than 1. **b,** Images of mice with NCI-N87 tumors at day 21 after drug treatment. Pre-established NCI-N87 tumor xenografts were treated with vehicle, trastuzumab (intraperitoneal, 10 mg/kg, 2 × /wk × 3), TFBG (intraperitoneal, 50 mg/kg, 1 × /day × 21), or a combination of the agents. **c,** Body weight changes of mice before and after drug treatment. Data are the mean ± s.e.m (n=8 independent mice per group). **d,** Tumor weight of NCI-N87 tumor xenografts mice treated as described in (b). Data are the mean ± s.e.m. The *p* values were determined by one-way ANOVA (n=8 independent mice per group). **e,** Images of mice with NCI-N87 tumors at day 21 after drug treatment. Pre-established NCI-N87 tumor xenografts were treated with vehicle, trastuzumab (intraperitoneal, 10 mg/kg, 2×/wk×3), TFBG (subcutaneous, 2.5 mg/kg, 1×/day×21), or a combination of the agents. **f and g,** Tumor volume (f) and weight (g) of NCI-N87 tumor xenograft mice treated as described in (e). Data are the mean ± s.e.m. The *p* values were determined by one-way ANOVA (n=8 independent mice per group). **h,** Co-immunoprecipitation assays of the Shc1-SHCBP1 interaction in cells treated with TFBG at the indicated concentrations. IP: immunoprecipitation; WCL: whole cell lysates. Data of **h** are representative of two independent experiments.

**Supplementary Table 1. The evaluation of prognostic factors for HER2 positive gastric cancer patients.**

| Variable | Univariate analysis | | | Multivariate analysis | | |
| --- | --- | --- | --- | --- | --- | --- |
|  | HR | 95% CI | *p* value | HR | 95% CI | *p* value |
| SHCBP1 H-score |  |  |  |  |  |  |
| ＜70 | 1 |  |  | 1 |  |  |
| ≥70 | 2.507 | 1.039-6.049 | 0.041 | 2.815 | 1.030-7.688 | 0.044 |
| Age at diagnosis (years) |  |  |  |  |  |  |
| ≤50 | 1 |  |  |  |  |  |
| >50 | 1.536 | 0.573-4.117 | 0.393 |  |  |  |
| Sex |  |  |  |  |  |  |
| male | 1 |  |  | 1 |  |  |
| female | 2.292 | 1.001-5.247 | 0.050 | 5.052 | 1.885-13.538 | 0.001 |
| Tumor location |  |  |  |  |  |  |
| Proximal stomach | 1 |  |  |  |  |  |
| Middle stomach | 0.679 | 0.196-2.350 | 0.541 |  |  |  |
| Distal stomach | 1.048 | 0.377-2.915 | 0.929 |  |  |  |
| Histological type |  |  |  |  |  |  |
| Diffuse | 1.186 | 0.468-3.008 | 0.719 |  |  |  |
| Intestinal | 1 |  |  |  |  |  |
| Mixed | 0.739 | 0.268-2.034 | 0.558 |  |  |  |
| Tumor size (cm) |  |  |  |  |  |  |
| ≥5 | 1 |  |  | 1 |  |  |
| ＜5 | 0.313 | 0.136-0.717 | 0.006 | 0.332 | 0.120-0.919 | 0.034 |
| Depth of invasion |  |  |  |  |  |  |
| T1 | 1 |  |  | 1 |  |  |
| T2-4 | 4.442 | 1.044-18.900 | 0.044 | 0.985 | 0.176-5.511 | 0.986 |
| Lymph node status |  |  |  |  |  |  |
| Negative | 1 |  |  | 1 |  |  |
| Positive | 4.140 | 1.639-10.462 | 0.003 | 3.037 | 0.930-9.912 | 0.066 |
| Clinical stage |  |  |  |  |  |  |
| Low (1) | 1 |  |  |  |  |  |
| High (2-4) | 2.533 | 0.945-6.787 | 0.065 |  |  |  |

H-score: Histo-score, HR: hazard ratio, CI: confidence interval. The *p* values were determined by cox proportional hazards regression models (n=60 independent samples).

**Supplementary Table 2. HER2-positive gastric cancer patients’ response to trastuzumab based on SHCBP1 levels.**

| ID | Age (year) | Sex | Stage | Differentiation | HER2 | SHCBP1 H-score | OS (months) | Treatment regimen |
| --- | --- | --- | --- | --- | --- | --- | --- | --- |
| 1 | 65 | M | 4b | medium | 3+ | 150 | 12.1 | trastuzumab + docetaxel |
| 2 | 67 | M | 4 | low-medium | 3+ | 120 | 17.2 | trastuzumab + tegafur, gimeracil and oteracil potassium |
| 3 | 68 | M | 4 | low-medium | 3+ | 80 | 3.0 | trastuzumab + oxaliplatin + capecitabine |
| 4 | 55 | F | 4 | low-medium | 3+ | 90 | 4.0 | trastuzumab + tegafur, gimeracil and oteracil potassium |
| 5 | 56 | M | 4 | low | 3+ | 180 | 4.0 | trastuzumab |
| 6 | 69 | F | 2a | high-medium | 3+ | 90 | 8.3 | trastuzumab + docetaxel + apatinib |
| 7 | 57 | M | 4 | low-medium | 3+ | 180 | 17.6 | trastuzumab + apatinib |
| 8 | 69 | M | 4 | low-medium | 3+ | 20 | 26.4 | trastuzumab + tegafur, gimeracil and oteracil potassium + paclitaxel |
| 9 | 63 | M | 3c | low-medium | 3+ | 10 | 18.0 | trastuzumab + docetaxel + nedaplatin |
| 10 | 76 | M | 4 | low-medium | 3+ | 30 | 15.3 | trastuzumab + oxaliplatin + tegafur, gimeracil and oteracil potassium |
| 11 | 67 | M | 4 | low-medium | 3+ | 60 | 12.1 | trastuzumab + capecitabine |
| 12 | 66 | F | 4 | low-medium | 3+ | 10 | 36.0 | trastuzumab + tegafur, gimeracil and oteracil potassium |
| 13 | 51 | M | 4 | low | 3+ | 30 | 8.6 | trastuzumab + docetaxel + tegafur, gimeracil and oteracil potassium |
| 14 | 69 | M | 4 | low-medium | 3+ | 120 | 36.0 | trastuzumab + oxaliplatin + tegafur, gimeracil and oteracil potassium |
| 15 | 64 | M | 4 | low-medium | 3+ | 50 | 36.0 | trastuzumab + capecitabine |
| 16 | 52 | M | 4 | medium | 3+ | 30 | 31.4 | trastuzumab + docetaxel + oxaliplatin |
| 17 | 72 | M | 4 | high-medium | 3+ | 20 | 24.3 | trastuzumab + capecitabine |
| 18 | 72 | M | 4 | low-medium | 3+ | 180 | 11.4 | trastuzumab + capecitabine |
| 19 | 58 | M | 4 | low-medium | 3+ | 120 | 4.4 | trastuzumab + capecitabine + oxaliplatin |
| 20 | 66 | M | 4 | low | 3+ | 30 | 3.0 | trastuzumab + oxaliplatin + tegafur, gimeracil and oteracil potassium |
| 21 | 65 | M | 4 | low-medium | 3+ | 150 | 5.7 | trastuzumab + oxaliplatin |
| 22 | 49 | M | 4 | medium | 3+ | 20 | 36.0 | trastuzumab + irinotecan (oxaliplatin) |

OS: overall survival; M: male; F: female; H-score: Histo-score.

**Supplementary Table 3. Sequences of shRNAs used.**

| Target | Sequences (5’-3’) |
| --- | --- |
| SHCBP1-shRNA2 | CCGGCCAATTACAGTGAGTCTGATTCTCGAGAATCAGACTCACTGTAATTGGTTTTTG |
| SHCBP1-shRNA3 | CCGGCTTGGTGAAACCTACAATCTTCTCGAGAAGATTGTAGGTTTCACCAAGTTTTTG |
| MISP-shRNA2 | CCGGCAGTCATCTGATCTGCTGGAACTCGAGTTCCAGCAGATCAGATGACTGTTTTTG |
| MISP- shRNA3 | CCGGGACACCAGCTACACATACCATCTCGAATGGTATGTGTAGCTGGTGTCTTTTTG |

**Supplementary Table 4. Primer sequences used for qRT-PCR.**

| Gene | Sequences (5’-3’) |
| --- | --- |
| GAPDH-Forward | GAAGGCTGGGGCTCATTT |
| GAPDH-Reverse | CAGGAGGCATTGCTGATGAT |
| SHCBP1-Forward | TGGCACACTAATGTGTTCAAGG |
| SHCBP1-Reverse | TCACAGAGGTATGGTTCAGCA |
| MISP-Forward | TCCCAGTCATCTGATCTGCTG |
| MISP-Reverse | ATCTGGCGTTGGGGAGAAGA |
| JUP-Forward | CTCTGTGCGTCTCAACTATGG |
| JUP-Reverse | GATCAAGCCGATGGTTGCCT |
| LYN-Forward | TGCAGAGGGAATGGCATACAT |
| LYN-Reverse | TGACTCGGAGACCAGAACATTAG |
| EPHA2-Forward | TGGCTCACACACCCGTATG |
| EPHA2-Reverse | GTCGCCAGACATCACGTTG |
| RASAL2-Forward | GACAGAAACACGAGCTTTCGG |
| RASAL2-Reverse | CATGGGAACGTGACTCTTTTAGT |
