# Supplementary material for: Hyperactivation of HER2-SHCBP1-PLK1 axis promotes tumor cell mitosis and impairs trastuzumab sensitivity to gastric cancer
Source: Nat Commun. 2021 May 14;12:2812. doi: 10.1038/s41467-021-23053-8 (PMC8121856; doi:10.1038/s41467-021-23053-8)
Supplement: Supplementary file 3 — Reporting Summary [file 41467_2021_23053_MOESM3_ESM.pdf]

nature research

Corresponding author(s): Zuoyi Jiao and Hui Sun

Last updated by author(s): Apr 1, 2021

Reporting Summary

Nature Research wishes to improve the reproducibility of the work that we publish. This form provides structure for consistency and transparency in reporting. For further information on Nature Research policies, see our Editorial Policies and the Editorial Policy Checklist.

Statistics

For all statistical analyses, confirm that the following items are present in the figure legend, table legend, main text, or Methods section.

n/a	Confirmed	
☐	☒	The exact sample size (*n*) for each experimental group/condition, given as a discrete number and unit of measurement
☐	☒	A statement on whether measurements were taken from distinct samples or whether the same sample was measured repeatedly
☐	☒	The statistical test(s) used AND whether they are one- or two-sided<br>*Only common tests should be described solely by name; describe more complex techniques in the Methods section.*
☐	☒	A description of all covariates tested
☐	☒	A description of any assumptions or corrections, such as tests of normality and adjustment for multiple comparisons
☐	☒	A full description of the statistical parameters including central tendency (e.g. means) or other basic estimates (e.g. regression coefficient) AND variation (e.g. standard deviation) or associated estimates of uncertainty (e.g. confidence intervals)
☐	☒	For null hypothesis testing, the test statistic (e.g. *F*, *t*, *r*) with confidence intervals, effect sizes, degrees of freedom and *P* value noted<br>*Give P values as exact values whenever suitable.*
☒	☐	For Bayesian analysis, information on the choice of priors and Markov chain Monte Carlo settings
☒	☐	For hierarchical and complex designs, identification of the appropriate level for tests and full reporting of outcomes
☒	☐	Estimates of effect sizes (e.g. Cohen's *d*, Pearson's *r*), indicating how they were calculated

Our web collection on statistics for biologists contains articles on many of the points above.

Software and code

Policy information about availability of computer code

Data collection	RT-PCR data was collected by a Real-Time PCR Detection System (LightCycle 96, Roche).<br>Cell viability was measured in the Microplate Reader (Multiskan FC System, Thermo-scientific).<br>For immunofluorescence staining and FRET assay, images were acquired by the confocal microscope (Zeiss LSM880, Germany).<br>Immunohistochemistry images were acquired with a KF-PRO-120 scanner (Konfoong, China).<br>Immunoblotting data was obtained by the imaging system (MiniChemi 610 plus, China).<br>Time-lapse imaging was collected by a time-lapse microscope system (Operetta CLS, PerkinElmer).<br>Flow cytometry data was acquired by CytExpert (version 2.4.0.28).<br>In vivo small animal imaging was conducted by an in vivo imaging system (Vieworks, Smart-LF, Korean).<br>Surface plasmon resonance (SPR) was conducted by Biacore (Biacore T200, GE Healthcare).<br>Microscale thermophoresis (MST) assays was conducted by Monolith NT.115 system (NanoTemper Technologies GmbH, German).<br>Inhibitor virtual screening was conducted by MOE software (version 2019) and Stardrop software (version 6.5.0).
Data analysis	SPSS (version 25.0) was used for data analysis; GraphPad Prism (version 8.0) was used for plotting and graphing; Zeiss LSM880 was used for FRET analysis; ModFit LT (version 5.0.9) was used for flow cytometry analysis; Microsoft excel 2019 was used for RT-PCR analysis; Image J (version 1.53c) was used for cell clones, immunofluorescence and immunohistochemistry images; CalcuSyn software (version 2.0) was used for combination index analysis; For figure 1b and 2d, R programming language (version 4.0.2), beeswarm (version 0.2.3), limma (version 3.44.3), sva (version 3.36.0) and OriginPro 2021 (version 9.8.0.200) were used.

For manuscripts utilizing custom algorithms or software that are central to the research but not yet described in published literature, software must be made available to editors and reviewers. We strongly encourage code deposition in a community repository (e.g. GitHub). See the Nature Research guidelines for submitting code & software for further information.

Data

Policy information about availability of data

All manuscripts must include a data availability statement. This statement should provide the following information, where applicable:

- Accession codes, unique identifiers, or web links for publicly available datasets
- A list of figures that have associated raw data
- A description of any restrictions on data availability

The expression profile microarray data of gastric cancer tissues in this study has been deposited in the ArrayExpress database under accession code E-MTAB-9990 (https://www.ebi.ac.uk/arrayexpress/experiments/E-MTAB-9990/). Published datasets are available from Oh et al. (GSE66229, [https://www.ncbi.nlm.nih.gov/geo/query/acc.cgi?acc=GSE66229]), Liu et al. (GSE54129, [https://www.ncbi.nlm.nih.gov/geo/query/acc.cgi?acc=GSE54129]), Cristescu et al. (GSE62254, [https://www.ncbi.nlm.nih.gov/geo/query/acc.cgi?acc=GSE62254]), Ooi et al. (GSE15459, [https://www.ncbi.nlm.nih.gov/geo/query/acc.cgi?acc=GSE15459]) and Lei et al. (GSE34942, [https://www.ncbi.nlm.nih.gov/geo/query/acc.cgi?acc=GSE34942]). The correlation of SHCBP1 expression with survival of HER2 positive or negative patients was analyzed on the website "http://kmplot.com/analysis/". Source data are provided with this paper.

Field-specific reporting

Please select the one below that is the best fit for your research. If you are not sure, read the appropriate sections before making your selection.

☒ Life sciences ☐ Behavioural & social sciences ☐ Ecological, evolutionary & environmental sciences

For a reference copy of the document with all sections, see nature.com/documents/nr-reporting-summary-flat.pdf

Life sciences study design

All studies must disclose on these points even when the disclosure is negative.

Sample size	No statistical methods were used to predetermine sample size. The sample sizes were determined based on the previous study (Yu, ZY. et al. Oncogene, 2021, 40(5): 1027-1042), and the sizes are large enough to ensure the credibility of results.
Data exclusions	No data was excluded
Replication	The experimental findings were reliably reproduced in two to three times independent experiments as indicated throughout the manuscript.
Randomization	For in vitro assays, cells were randomly assigned to either experiment or control group. A biological sample was split into the equal parts for control and experiment groups.<br>Mice were randomly divided into control and experimental groups at the start of each experiment.
Blinding	Investigators were not blinded to the identity of samples to ensure appropriate data collection and because experimental results are quantitative in nature, not readily subject to investigator bias. To ensure consistent experimental conditions, all control and experimental samples were processed in parallel. However, all the data analyses were performed by blinded scientists.

Reporting for specific materials, systems and methods

We require information from authors about some types of materials, experimental systems and methods used in many studies. Here, indicate whether each material, system or method listed is relevant to your study. If you are not sure if a list item applies to your research, read the appropriate section before selecting a response.

Materials & experimental systems

n/a	Involved in the study	
☐	☒	Antibodies
☐	☒	Eukaryotic cell lines
☒	☐	Palaeontology and archaeology
☐	☒	Animals and other organisms
☐	☒	Human research participants
☒	☐	Clinical data
☒	☐	Dual use research of concern

Methods

n/a	Involved in the study	
☒	☐	ChIP-seq
☐	☒	Flow cytometry
☒	☐	MRI-based neuroimaging

Antibodies

Antibodies used	Rabbit monoclonal anti-SHC, Abcam, ab33770, Lot. 00044779, 1:100 for IF<br>Rabbit polyclonal anti-SHCBP1, Sigma, HPA048876, Lot. 00062242, 1:100 for IF, 1:200 for IHC<br>Rabbit polyclonal anti-SHCBP1, Proteintech, 12672-1-AP, Lot. 00091299, 1:1000 for WB<br>Rabbit monoclonal anti-ERBB2, Abcam, ab214275, Lot. GR3283213-4, 1:1000 for WB, 1:100 for IF, 1:200 for IHC

Rabbit monoclonal anti-Phospho-ERBB2, Cell signaling, 2243, Lot. 12, 1:1000 for WB
Rabbit polyclonal anti-MISP, Abcam, ab254919, Lot. GR3306502-1, 1:1000 for WB, 1:100 for IF
Rabbit polyclonal anti-PLK1, Proteintech, 10305-1-AP, Lot. 00060709, 1:1000 for WB, 1:100 for IF
Mouse monoclonal anti-α-Tubulin, Proteintech, 66031-1-Ig, Lot. 10004185, 1:100 for IF
Rabbit monoclonal anti-γ-tubulin, Abcam, ab179503, Lot. GR208789-8, 1:100 for IF
Rabbit polyclonal anti-HA, Invitrogen, 71-5500, Lot. UD280764, 1:1000 for WB
Rabbit polyclonal anti-Flag, Sigma, SAB4301135, Lot. SLCC6485, 1:1000 for WB, 1:100 for IF
Mouse monoclonal anti-β-actin, Proteintech, 66009-1-Ig, Lot. 10004156, 1:1000 for WB
Rabbit polyclonal anti-GAPDH, Proteintech, 10494-1-AP, Lot. 00083125, 1:1000 for WB
Rabbit polyclonal anti-Lamin B1, Proteintech, 12987-1-AP, Lot. 00084411, 1:1000 for WB
Rabbit polyclonal anti-Phosphoserine, Abcam, ab9332, Lot. GR3179704-23, 1:500 for WB
Rabbit monoclonal anti-Thiophosphate ester, Abcam, ab92570, Lot. GR237393-15, 1:5000 for WB
Rabbit monoclonal anti-Ki67, Abcam, ab156956, Lot. GR271158-29, 1:200 for IHC
Goat polyclonal anti-mouse Alexa Fluor 488-conjugated antibody, Abcam, ab150113, Lot. GR3353891-1, 1:400 for IF
Goat polyclonal anti-rabbit Alexa Fluor 647-conjugated antibody, Abcam, ab150079, Lot. GR3312382-2, 1:400 for IF
Peroxidase-conjugated AffiniPure Goat Anti-mouse IgG (H+L), Bioworld, BS12478, Lot. AA102012, 1:10000 for WB
Peroxidase-conjugated AffiniPure Goat Anti-rabbit IgG (H+L), Bioworld, BS13278, Lot. AA112027, 1:10000 for WB
anti-Flag M2 affinity gel, Sigma, A2220, Lot. SLBZ1501

Validation

All the antibodies used in the study were commercial and validated by the manufactures.
ab33770 (SHC): The antibody has been referenced in 7 researches and used for immunohistochemistry and immunobloting specifically. More information about SHC antibody can be found on the website: https://www.abcam.com/shc-antibody-ep332y-ab33770.html.
HPA048876 (SHCBP1): The antibody has been referenced in 3 researches and used for immunohistochemistry, immunofluorescence and immunobloting specifically. More information about SHCBP1 antibody can be found on the website: https://www.sigmaaldrich.com/catalog/product/sigma/hpa048876?lang=zh&region=CN.
12672-1-AP (SHCBP1): The antibody has been referenced in 5 researches and used for immunohistochemistry, immunofluorescence and immunobloting specifically. More information about SHCBP1 antibody can be found on the website: https://www.ptgcn.com/products/SHCBP1-Antibody-12672-1-AP.htm.
ab214275 (ERBB2): The antibody has been referenced in 2 researches and used for immunoprecipitation, immunohistochemistry and immunobloting specifically. More information about ERBB2 antibody can be found on the website: https://www.abcam.com/erbb2--her2-antibody-epr19547-12-ab214275.html.
2243S (Phospho-ERBB2): The antibody has been referenced in 120 researches and used for immunoprecipitation, immunohistochemistry, immunobloting and ELISA specifically. More information about Phospho-ERBB2 antibody can be found on the website: https://www.cellsignal.com/products/primary-antibodies/phospho-her2-erbb2-tyr1221-1222-6b12-rabbit-mab/2243;jsessionid=-fjZQp6Nz8O14rG4Mn5pvwADgWgZe8m1Z2G6lpYC?site-search-type=Products&N=4294956287&Ntt=2243s&fromPage=plp&_requestid=889141.
ab254919 (MISP): The antibody has been used for immunohistochemistry and immunobloting specifically. More information about MISP antibody can be found on the website: https://www.abcam.com/c19orf21-antibody-ab254919.html.
10305-1-AP (PLK1): The antibody has been referenced in 9 researches and used for immunofluorescence, immunohistochemistry and immunobloting specifically. More information about PLK1 antibody can be found on the website: https://www.ptglab.com/products/PLK1-Antibody-10305-1-AP.htm.
66031-1-Ig (α-Tubulin): The antibody has been referenced in 253 researches and used for immunoprecipitation, immunofluorescence, immunohistochemistry and immunobloting specifically. More information about α-Tubulin antibody can be found on the website: https://www.ptglab.com/products/tubulin-Alpha-Antibody-66031-1-Ig.htm.
ab179503 (γ-tubulin): The antibody has been referenced in 10 researches and used for immunoprecipitation, immunohistochemistry and immunobloting specifically. More information about γ-tubulin antibody can be found on the website: https://www.abcam.cn/gamma-tubulin-antibody-epr16793-ab179503.html.
71-5500 (HA): The antibody has been referenced in 73 researches and used for immunoprecipitation, immunofluorescence, immunohistochemistry and immunobloting specifically. More information about HA antibody can be found on the website: https://www.thermofisher.com/cn/zh/antibody/product/HA-Tag-Antibody-clone-SG77-Polyclonal/71-5500.
SAB4301135 (Flag): The antibody has been referenced in 21 researches and used for immunoprecipitation, immunofluorescence and immunobloting specifically. More information about Flag antibody can be found on the website: https://www.sigmaaldrich.com/catalog/product/sigma/sab4301135?lang=zh&region=CN.
66009-1-Ig (β-actin): The antibody has been referenced in 1511 researches and used for immunoprecipitation, immunofluorescence, immunohistochemistry and immunobloting specifically. More information about β-actin antibody can be found on the website: https://www.abcam.com/beta-actin-antibody-ab8227.html.
10494-1-AP (GAPDH): The antibody has been referenced in 2116 researches and used for immunoprecipitation, immunofluorescence, immunohistochemistry and immunobloting specifically. More information about GAPDH antibody can be found on the website: https://www.ptglab.com/products/GAPDH-Antibody-10494-1-AP.htm.
12987-1-AP (Lamin B1): The antibody has been referenced in 398 researches and used for immunoprecipitation, immunofluorescence, immunohistochemistry and immunobloting specifically. More information about Lamin B1 antibody can be found on the website: https://www.ptglab.com/products/LMNB1-Antibody-12987-1-AP.htm.
ab9332 (Phosphoserine): The antibody has been referenced in 98 researches and used for immunoprecipitation, immunobloting and ELISA specifically. More information about Phosphoserine antibody can be found on the website: https://www.abcam.com/phosphoserine-antibody-ab9332.html.
ab92570 (Thiophosphate ester): The antibody has been referenced in 55 researches and used for immunobloting specifically. More information about Thiophosphate ester antibody can be found on the website: https://www.abcam.com/thiophosphate-ester-antibody-51-8-ab92570.html.
ab156956 (Ki67): The antibody has been referenced in 20 researches and used for immunohistochemistry and immunobloting specifically. More information about Ki67 antibody can be found on the website: https://www.abcam.com/ki67-antibody-oti5d7-ab156956.html.

ab150113 (Alexa Fluor@488): The antibody has been referenced in 322 researches and used for immunohistochemistry and ELISA specifically. More information about Alexa Fluor@488 antibody can be found on the website: https://www.abcam.com/goat-mouse-igg-hl-alexa-fluor-488-ab150113.html.
ab150079 (Alexa Fluor@647): The antibody has been referenced in 126 researches and used for immunohistochemistry and ELISA specifically. More information about Alexa Fluor@647 antibody can be found on the website: https://www.abcam.com/goat-rabbit-igg-hl-alexa-fluor-647-ab150079.html.
BS12478 (IgG): The antibody has been used for immunohistochemistry and immunobloting specifically. More information about IgG secondary antibody can be found on the website: https://www.bioworlde.com/Goat-anti-Mouse-IgG-(H+L)--HRP(BS12478).html.
BS13278 (IgG): The antibody has been used for immunohistochemistry and immunobloting specifically. More information about IgG secondary antibody can be found on the website: https://www.bioworlde.com/Goat-anti-Rabbit-IgG-(H+L)-HRP(BS13278).html.
A2220 (anti-Flag M2 affinity gel): The gel beads has been used for Co-IP specifically. More information about anti-Flag M2 affinity gel can be found on the website: https://www.sigmaaldrich.com/catalog/product/sigma/a2220?lang=zh&region=CN.

Eukaryotic cell lines

Policy information about cell lines

Cell line source(s)	Human gastric cancer cells NCI-N87, HGC-27, MKN-45 and AGS were obtained from the institute of Basic Medical Sciences, Chinese Academy of Medical Sciences (CAMS, Beijing, China). Human gastric cancer cells KATO-II, and HS-746T were obtained from the Kunming institute of zoology, Chinese Academy of Sciences (CAS, Kunming, China). HEK293T cells were obtained from the ATCC, and human gastric cancer cells SNU-216 were obtained from the Korean Cell Line Bank.
Authentication	All cell lines have been validated by short tandem repeat (STR) DNA fingerprinting using the commercially available EX20 Kit from AGCU.
Mycoplasma contamination	All cell lines have been tested negative for mycoplasma contamination.
Commonly misidentified lines (See ICLAC register)	No misidentified cell lines were used in the study.

Animals and other organisms

Policy information about studies involving animals; ARRIVE guidelines recommended for reporting animal research

Laboratory animals	6 to 8-week-old female BALB/C Nude mice were housed at 18-25 °C with 40-70% humidity, maintained on a 12h light/dark cycle and provided with food and water ad libitum in individually ventilated units (Techniplast) in specific pathogen-free facilities at the cuiying biomedical research center of Lanzhou University Second Hospital, China.
Wild animals	The study did not involve wild animals.
Field-collected samples	The study did not involve samples collected from the field.
Ethics oversight	The study in the mice was approved by the Animal Ethics Committee of Lanzhou University Second Hospital.

Note that full information on the approval of the study protocol must also be provided in the manuscript.

Human research participants

Policy information about studies involving human research participants

Population characteristics	All the human participants are adult male or female and aged between 25-76 years old. Samples were collected from patients diagnosed with gastric cancer, that had not been treated yet. For HER2-positive gastric cancer patients, trastuzumab-based therapy was provided. All tissues were obtained from Lanzhou University Second Hospital.
Recruitment	Participants were recruited as part of routine clinical patients undergoing gastroscopy biopsy or postoperative pathology. Self-selection bias is unlikely to have affected the results.
Ethics oversight	Medical Ethics Review Board at the Lanzhou University Second Hospital.

Note that full information on the approval of the study protocol must also be provided in the manuscript.

Flow Cytometry

Plots

Confirm that:

- [x] The axis labels state the marker and fluorochrome used (e.g. CD4-FITC).
- [x] The axis scales are clearly visible. Include numbers along axes only for bottom left plot of group (a 'group' is an analysis of identical markers).
- [x] All plots are contour plots with outliers or pseudocolor plots.
- [x] A numerical value for number of cells or percentage (with statistics) is provided.

Methodology

Sample preparation	Flow cytometry analysis was performed using an ModFit LT software. Briefly, cells were collected and fixed with precooled 75% ethanol overnight at 4 °C. The cells were submersed in 37 °C water for 30 min, followed by washing with PBS buffer twice and blocked in PI/RNase staining buffer (BD 550825) for 15 minutes at room temperature according to manufacturer instruction.
Instrument	CytoFLEX (Beckman Coulter)
Software	CytExpert (version 2.4.0.28) for data collection, ModFit LT (version 5.0.9) for data analysis
Cell population abundance	For each sample, flow cytometry data for 10000 events were collected. After gating, at least 50% of events were identified as singlets
Gating strategy	Cells were first gated based on forward scatter (FSC) and side scatter (SSC) to remove debris while preserving cells based on size. Singlets were then gated cells (FSC-A vs. SSC-A and FL2-A PE-A vs. FL2-H PE-H).

- [x] Tick this box to confirm that a figure exemplifying the gating strategy is provided in the Supplementary Information.
